# Neural Progenitor Cells Undergoing Yap/Tead-Mediated Enhanced Self-Renewal Form Heterotopias More Easily in the Diencephalon than in the Telencephalon

**DOI:** 10.1007/s11064-017-2390-x

**Published:** 2018-01-01

**Authors:** Kanako Saito, Ryotaro Kawasoe, Hiroshi Sasaki, Ayano Kawaguchi, Takaki Miyata

**Affiliations:** 1 Department of Anatomy and Cell Biology, Nagoya University Graduate School of Medicine, 65 Tsurumai, Showa, Nagoya, 466-8550 Japan; 2 Laboratory for Embryogenesis, Graduate School of Frontier Biosciences, Osaka University, 1-3 Yamadaoka, Suita, Osaka, 565-0871 Japan

**Keywords:** Neural progenitor cells, Ventricular zone, Self-renewal, Yap, Tead, Heterotopia

## Abstract

Spatiotemporally ordered production of cells is essential for brain development. Normally, most undifferentiated neural progenitor cells (NPCs) face the apical (ventricular) surface of embryonic brain walls. Pathological detachment of NPCs from the apical surface and their invasion of outer neuronal territories, i.e., formation of NPC heterotopias, can disrupt the overall structure of the brain. Although NPC heterotopias have previously been observed in a variety of experimental contexts, the underlying mechanisms remain largely unknown. Yes-associated protein 1 (Yap1) and the TEA domain (Tead) proteins, which act downstream of Hippo signaling, enhance the stem-like characteristics of NPCs. Elevated expression of Yap1 or Tead in the neural tube (future spinal cord) induces massive NPC heterotopias, but Yap/Tead-induced expansion of NPCs in the developing brain has not been previously reported to produce NPC heterotopias. To determine whether NPC heterotopias occur in a regionally characteristic manner, we introduced the Yap1-S112A or Tead-VP16 into NPCs of the telencephalon and diencephalon, two neighboring but distinct forebrain regions, of embryonic day 10 mice by in utero electroporation, and compared NPC heterotopia formation. Although NPCs in both regions exhibited enhanced stem-like behaviors, heterotopias were larger and more frequent in the diencephalon than in the telencephalon. This result, the first example of Yap/Tead-induced NPC heterotopia in the forebrain, reveals that Yap/Tead-induced NPC heterotopia is not specific to the neural tube, and also suggests that this phenomenon depends on regional factors such as the three-dimensional geometry and assembly of these cells.

## Introduction

Undifferentiated neural progenitor cells (NPCs) are central to the production of a variety of cells in developing and mature brains [1–9]. During the early embryonic period before neurons emerge, the nuclei/somata of NPCs are diffusely distributed throughout the initial wall of brain primordia [also called the neuroepithelium (NE)]. This is because NPCs, which adopt an elongated (~100 μm) epithelial morphology, move their nuclei/somata along the apicobasal axis in a cell cycle-dependent manner, a process called interkinetic nuclear migration (IKNM) [reviewed in 10, 11]. Horizontal assembly of NPCs individually differing in cell-cycle phase and nuclear/somal position makes this NE structure “pseudostratified” (i.e., although there are several layers of nuclei in the NE wall, one layer of cells can host multiple layers of nuclei). As the brain walls thicken (>100 μm), a new zone in which differentiated neurons accumulate is added outside (i.e., basal to) the original NE region, which is subsequently called the ventricular zone (VZ) (~100 μm), and NPCs elongate along the apicobasal axis by adding basal processes [4–6, 8–11]. Throughout the neuron-producing period, the elongated NPCs (also referred to as radial glial cells) keep their nuclei/somata in the apical (inner) part of the wall, as in the earlier NE period, maintaining the pseudostratified structure and VZ thickness, which correspond to the range of IKNM along the apicobasal axis. NPCs’ nuclei/somata move only in the VZ, and therefore do not intermingle with neurons located basally. Under diverse experimental and pathological conditions, however, NPCs’ nuclei/somata assume heterotopic positions; i.e., they invade neuronal territory ~200 μm away from the apical surface. A major reason for the formation of such NPC heterotopias is over-proliferation in the NE/VZ. For example, excessive activation of canonical Wnt-β-catenin signaling [12–14], FGF signaling [15], or cyclin D1 [16, 17], which can induce NPCs to maintain stem-like proliferative and undifferentiated status rather than undergoing quiescence and differentiation, results in extra-VZ nuclear/somal distribution of NPCs, and these mechanisms may underlie the pathogenesis of congenital brain diseases in humans [18, 19]. Furthermore, physiological dislocation of NPCs’ nuclei/somata to non-VZ positions seems to have accompanied neocortical evolution [8, 9, 20–23]. Thus, elucidation of the mechanisms that regulate the extra-VZ positioning of NPCs’ nuclei/somata is a critical goal in the field of developmental neuroscience.

A previous study that focused on the developing neural tube (designated here as the future spinal cord, not including the regions that form the future brain) showed that excessive activation of the Yes-associated protein 1 (Yap1) and the TEA domain (Tead) proteins, key DNA-binding platforms for Yap, in the NE results in over-proliferation of NPCs [24]. Yap1 and Tead are involved in cell density-dependent regulation of proliferation. At high cell densities, strong Hippo signaling suppresses cell proliferation by inhibiting nuclear Yap accumulation, whereas at low cell densities, weak Hippo signals allow nuclear Yap accumulation, promoting cell proliferation through Tead activation [25–27]. The Yap/Tead-induced over-proliferation or expansion of self-renewing stem-like NPCs in the neural tube NE/VZ is almost always accompanied by NPC heterotopias outside the VZ [24]. Interestingly, although Yap/Tead-induced NE/VZ expansion has been consistently reproduced in multiple studies [28–32], NPC heterotopias have not, to our knowledge, been clearly observed in primordial CNS regions other than the neural tube.

Hence, we sought to determine whether Yap/Tead-induced NPC heterotopias occur in a regionally characteristic manner. Although it is possible that NPC heterotopias might arise in a manner dependent upon the molecularly regulated regional identities of NPCs, it is also possible that the occurrence of NPC heterotopias reflects region-specific three-dimensional conditions (e.g., the geometry and assembly of NPCs). Yap1 and Tead play multiple roles in response to different physical/mechanical factors, such as tissue tension or compression [33–38], and they are involved in the epithelial–mesenchymal transition [26, 27, 39].

**Fig. 1:**
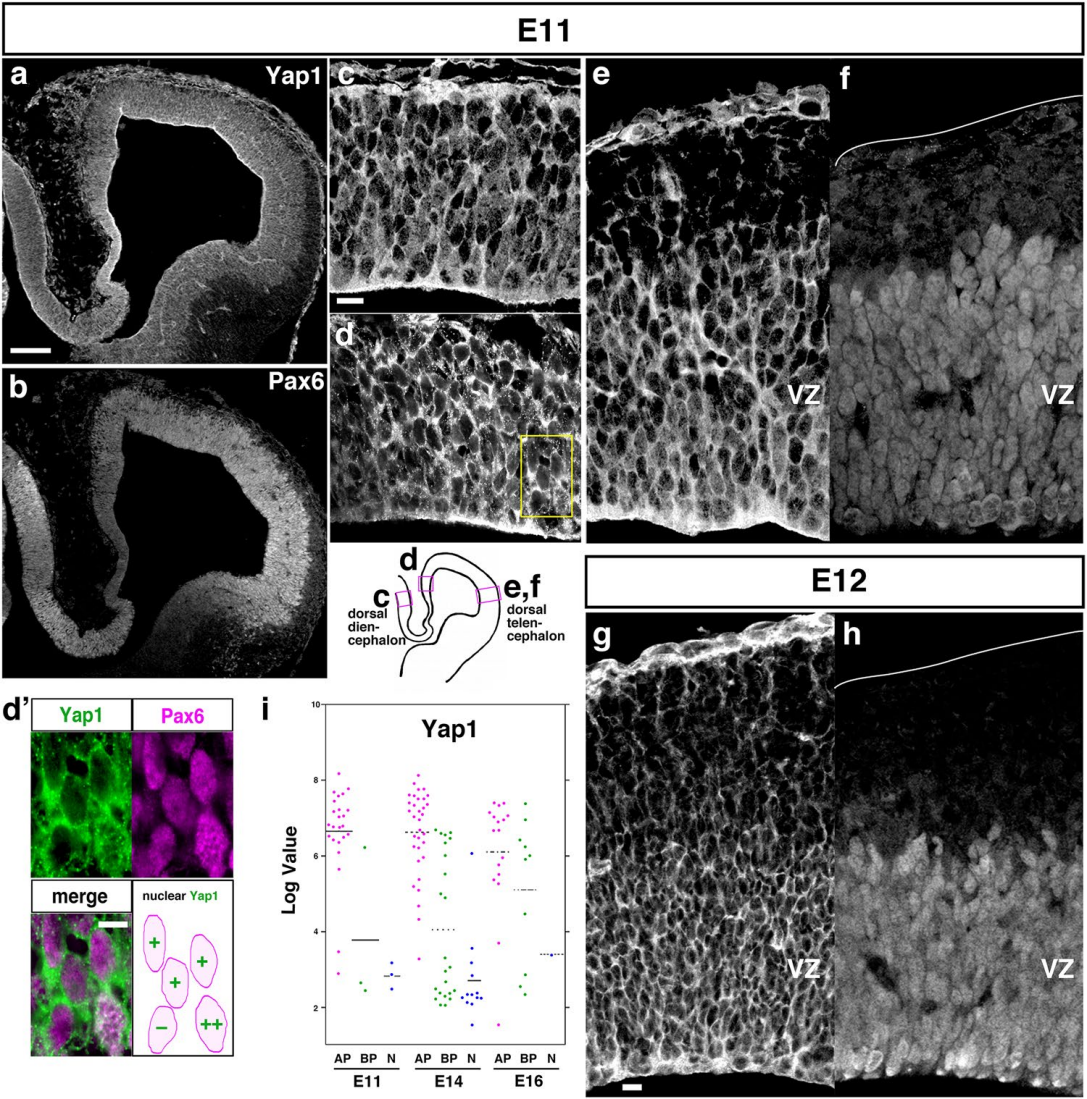
Yap1 is expressed in undifferentiated forebrain NPCs. A coronal section of an E11 mouse forebrain immunostained with anti-Yap1 (**a**) and anti-Pax6 (**b**). Yap1 immunoreactivity was observed throughout the pseudostratified NE of the dorsal diencephalon (**c**) and the medial telencephalic wall (**d**), and it was also seen in the similarly pseudostratified VZ of the lateral telencephalic wall (**e**). Outside the VZ, where Pax6 was not detected (**f**), radially elongated fibers (i.e., basal processes of NPCs) were also Yap1-positive, whereas neurons dominating in that region were Yap1-negative. NPCs showed nuclear anti-Yap1 staining, with some heterogeneity in the NE/VZ (**d’**). Similar immunostaining patterns were reproduced at E12 (**g, h**). Single-cell transcriptome analysis of E11, E14, and E16 telencephalic cells (**i**) revealed that *Yap1* expression was strongest in undifferentiated NPCs [designated as apical progenitors (AP)] and weakest in neurons (N), with intermediate levels of expression in basal progenitors (BP) differentiating while keeping mitotic activity [43, 44]. At E11, the NE/VZ was almost fully occupied by AP-type cells (undifferentiated NPCs). *Scale bars* 100 µm in **a** and **b**, 10 µm in **c**–**f**, **g**, and **h**, 5 µm in **d’**

To address this issue, we took advantage of in utero electroporation (IUE)-mediated gene transfer into the early embryonic mouse dorsal forebrain, which consists of the telencephalon (future cerebrum) and the dorsal diencephalon (future thalamus). The developing telencephalon and diencephalon share some common molecular hallmarks (e.g., Pax6 expression in the NE/VZ of both regions, Fig. 1). Morphologically, however, the extent to which a given wall portion (and NE/VZ contained) expands tangentially/laterally during the embryonic period differs between these regions. The dorso-ventral extension of the thalamic wall is clearly smaller than that of the cerebral wall, which expands to form a large vesicle/hemisphere [40]. These differences in physical limitations (inversely, lateral expandability) might underlie the differential susceptibility to experimental/pathological perturbations [41]. To investigate this possibility, we performed IUE at embryonic day 10 (E10), because (1) the forebrain primordium at this early stage is similar to the neural tube in terms of the composition and structure of the NE, and (2) in previous work, the use of the same protocol to activate Wnt3a induced mild NPC heterotopias in the telencephalon [14], allowing us to comparatively address possible contributions of Yap/Tead in NPC heterotopia formation. We found that artificial activation of Yap/Tead enhanced NPC self-renewal in both telencephalic and diencephalic NE/VZ, whereas NPC heterotopias formed much more frequently in the diencephalon than in the telencephalon.

## Materials and Methods

### Animals

Pregnant ICR mice were obtained from SLC (Japan). All protocols for animal experiments were approved by The Animal Care and Use Committee of the Nagoya University.

### Immunohistochemistry

Embryonic mouse brains were fixed by the periodate–lysine–paraformaldehyde (PLP) fixative, immersed in 20% sucrose, embedded in OCT compound (Miles, Elkhart, IN, USA), and then frozen and sectioned coronally (16 μm), as previously described [6, 14, 42]. Frozen sections were treated with the following primary antibodies: anti-Yap 1 (mouse, Abnova, Taipei, Taiwan); anti-Pax6 (rabbit, COVANCE, CA, USA); anti-BrdU (rat, Novus Biologicals, CO, USA); anti-P27 (mouse, BD Biosciences, NJ, USA); anti-βIII-tubulin (TUJ1) (mouse, COVANCE, CA, USA); or anti-GFP (rat, Nacalai Tesque, Kyoto, Japan; rabbit, MBL, Nagoya, Japan; chick, Aves Labs, OR, USA). After washes, sections were treated with Alexa Fluor 488–, Alexa Fluor 546–, or Alexa Fluor 647–conjugated secondary antibodies (Life Technologies), and subjected to confocal microscopy (Olympus FV1000, Tokyo, Japan).

### *Yap1* Expression in Single Cell Transcriptome Profiles

Information of *Yap1* expression levels in single E11, E14 and E16 telencephalic cells was obtained from the single cell transcriptome profiles available in the GEO database under accession codes: GSE10881 (for E14) and GSE55981 (for E11, E16) with probe set ID: 1448363_at.

### Plasmids

The Yap1-S112A mutant (pEF1α-HA-Yap-SA-IRES-EGFP), or Tead2-VP16 (pEF1α-Tead2-VP16-IRES-EGFP, encoding a fusion protein of the N-terminal region of Tead2 containing the TEA domain and the activation domain of herpes simplex virus VP16) was constructed using pMYs-HA-YAP-SA-IRES-EGFP and pMYs-Tead2-VP16-IRES-EGFP [26]. Changing the Ser 112 into Ala increases nuclear Yap1 and enhances proliferation beyond normal confluency [26]. It has also been known that increasing Tead activity by expressing the activator-modified Tead2 (Tead2-VP16) promoted cell proliferation beyond confluence and resulted in a higher saturation density [26]. As a control vector, pEF1α-IRES-EGFP was used.

### In Utero Electroporation

In utero electroporation (IUE) was performed using pregnant ICR mice at E10 as described previously [14]. DNA solution was injected into the lateral ventricle. The head of the embryo in the uterus was placed between the discs of a forceps-type electrode (disc electrodes of 1 mm; CUY560P1, NEPA GENE, Chiba, Japan), and electric pulses (50 V) were charged four times, resulting in gene transfection into the cerebral wall.

### In Vivo Comparison of Stem-Like Characteristics of NPCs

For obtaining %Pax6^+^/GFP^+^, embryos electroporated at E10 were fixed at E13. Frozen coronal sections were double immunostained with anti-GFP and anti-Pax6. For obtaining %Pax6^+^/GFP^+^BrdU^+^ or %p27^+^/GFP^+^BrdU^+^, embryos electroporated at E10 were labeled at E11 with bromodeoxyuridine (BrdU) through intra-peritoneal injection into mother mice (50 μg/g body weight), and fixed at E12. Frozen coronal sections were triple immunostained with ant-GFP, anti-BrdU, and anti-p27 or anti-Pax6, as previously described [14].

**Fig. 2:**
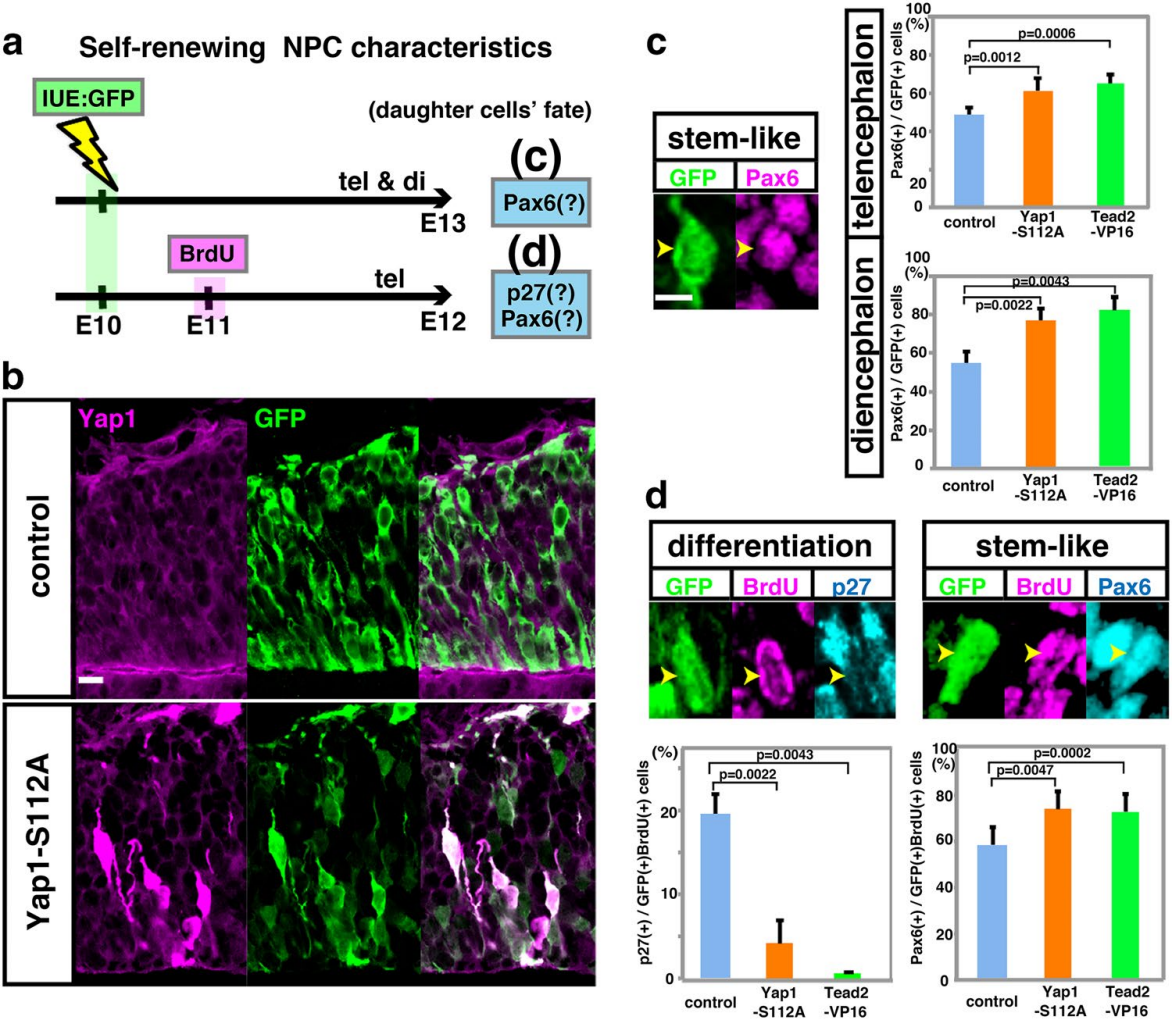
IUE-mediated expression of Yap1-S112A and Tead2-VP16 enhanced stem-like characteristics in telencephalic and diencephalic NPCs, as determined by protocols shown in **a**. Anti-Yap1 immunoreactivity increased in Yap-S112A–electroporated VZ cells (**b**). The proportion of total GFP^+^ cells that were also positive for Pax6 was greater in Yap1-S112A or Tead2-VP16 IUE cases in both telencephalon and diencephalon (**c**). The proportion of GFP^+^BrdU^+^ cells that were negative for p27 or positive for Pax6 was greater in Yap1-S112A– or Tead-VP16–electroporated diencephalic NPCs (**d**). *Scale bars* 10 µm in **b**, 5 µm in **c** and **d**

Total number of sections examined: for Pax6 assay at E13 in telencephalon (Fig. 2c), 7 for control, 6 for Yap1-S112A, and 7 for Tead2-VP16 (in each section, 44–107 cells were counted). For Pax6 assay at E13 in diencephalon (Fig. 2c), 6 for control, 6 for Yap1-S112A, and 5 for Tead2-VP16 (in each section, 61–159 cells were counted). For Pax6 assay at E12 in diencephalon (Fig. 2d), 13 for control, 7 for Yap1-S112A, and 18 for Tead2-VP16 (in each section, 28–158 cells were counted). For p27 assay in diencephalon (Fig. 2d), 6 for control, 6 for Yap1-S112A, and 5 for Tead2-VP16 (in each section, 10–41 cells were counted). These sections were prepared from three independent embryos in each of the control, Yap1-S112A, and Tead2-VP16 experiments.

### Statistical Analysis

**Fig. 3:**
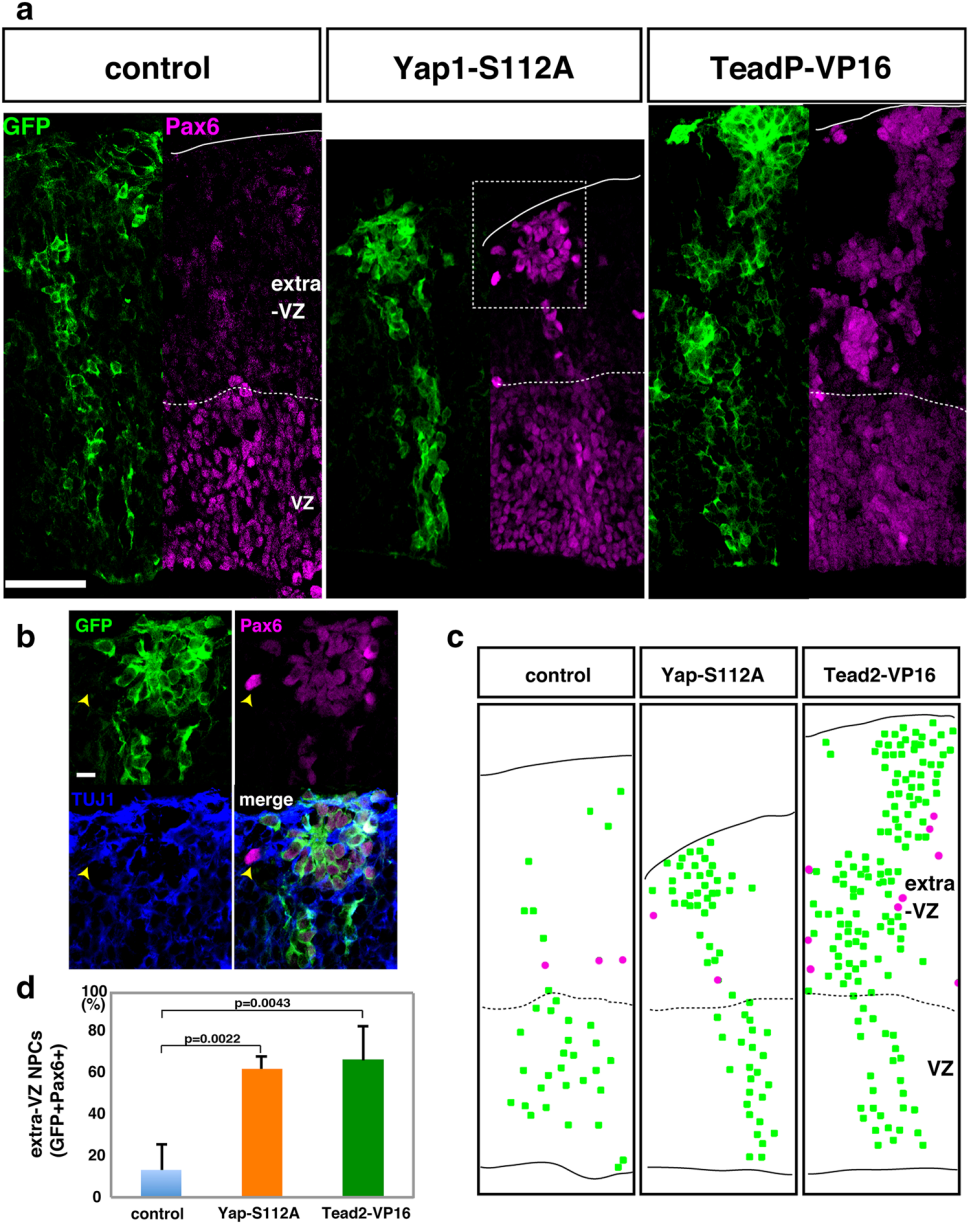
Yap/Tead overexpression in the dorsal diencephalon resulted in frequent and massive NPC heterotopias. **a** Anti-GFP and anti-Pax6 photomicrographs of E13 dorsal diencephalons that had been electroporated at E10 with the control, Yap1-S112A, or Tead2-VP16 vectors. **b** Basal zone dominated by TUJ1^+^ neurons in a Yap1-S112A-IUE telencephalic wall is shown at a higher magnification, containing a large cluster of heterotopic NPCs. Distribution of Pax6^+^ NPCs in **a** is schematically shown in **c** (*green* = GFP^+^Pax6^+^, *magenta* = GFP^−^Pax6^+^). Graph **d** summarizes the distribution of GFP^+^Pax6^+^ obtained from multiple sections [n = 4 for control, n = 5 for Yap1-S112A, and n = 4 for Tead2-VP16 (three independent embryos in each group)], showing that Yap1-S112A or Tead2-VP16 IUE induced massive NPCs heterotopias. In addition to GFP^+^Pax6^+^ cells predominantly constituting heterotopic clusters, some GFP^−^Pax6^+^ cells (*arrow* in *panel b, magenta* in *panel c*) were observed in or near the heterotopic clusters found in the basal-most portion of the telencephalic wall. *Scale bars* 50 µm in **a**, 10 µm in **b**

**Fig. 4:**
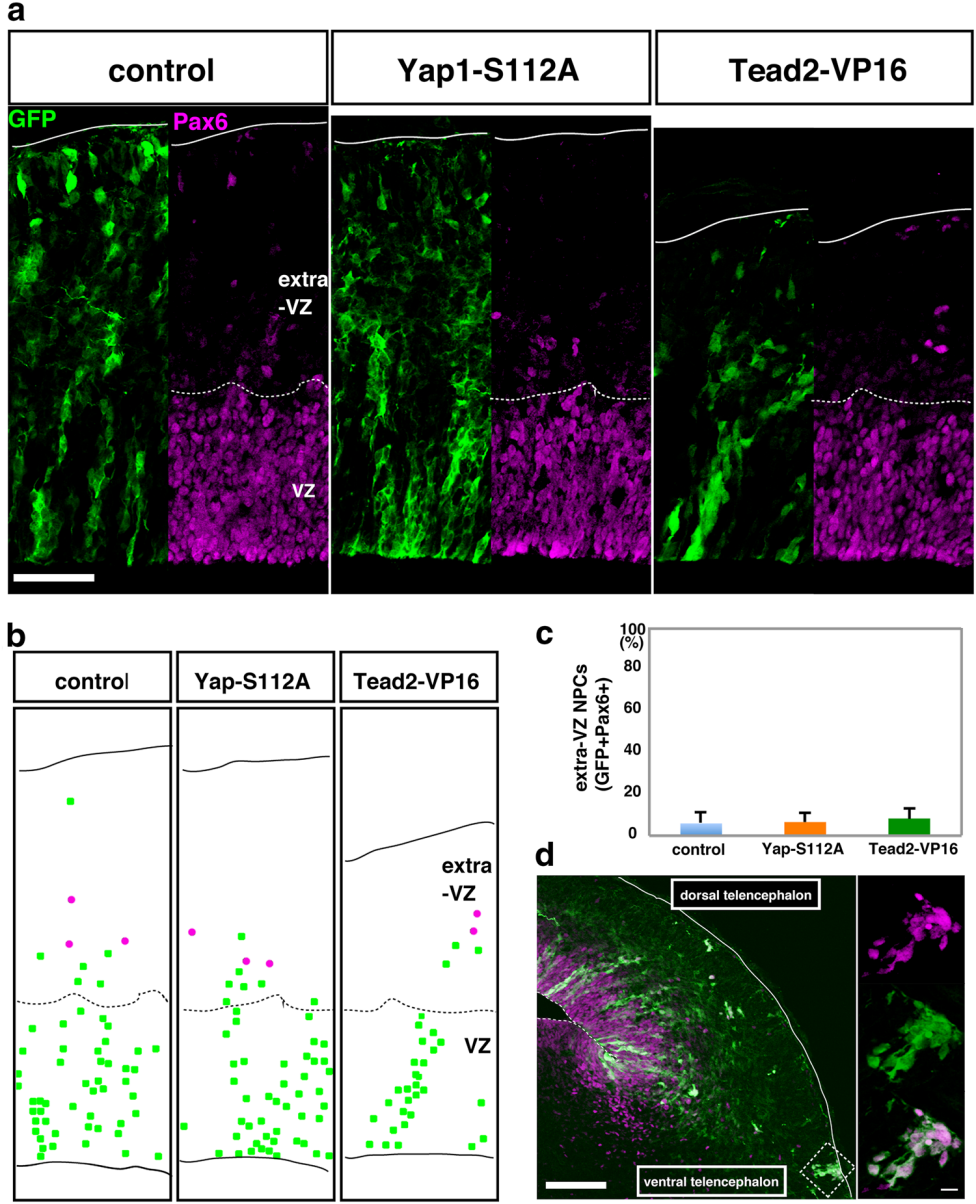
Yap1/Tead overexpression in the dorsal telencephalic VZ resulted only rarely in NPC heterotopias in the ventral-most region. **a** Anti-GFP and anti-Pax6 photomicrographs of E13 dorsal telencephalons that had been electroporated at E10 with the control, Yap1-S112A, or Tead2-VP16 vectors. Distribution of Pax6^+^ NPCs in **a** is schematically shown in **b** (*green* = GFP^+^Pax6^+^, *magenta* = GFP^−^Pax6^+^). Graph **c** summarizes the distribution of GFP^+^Pax6^+^ obtained from multiple sections [n = 5 for control, Yap1-S112A, and Tead2-VP16 (three independent embryos in each group)], showing that most GFP^+^Pax6^+^ NPCs were in the VZ. **d** An exceptional case in which a relatively large cluster of heterotopic GFP^+^Pax6^+^ NPCs was formed by Yap1-S112A IUE of the ventral-most part of the dorsal telencephalic VZ. Note that this region is physically constrained by a hairpin-loop bending of the VZ upon transition to the ventral telencephalon. *Scale bars* 50 µm in **a**, 100 µm (*left panels*) and 10 µm (*right panels*) in **d**

Differences were analyzed using Wilcoxon rank-sum test (in Figs. 2c, 3d, 4c) or Permuted Brunner Munzel test (in Fig. 2d) by R, and data were expressed as means ± SD.

## Results and Discussion

### Yap1 is Expressed in Early Embryonic Mouse Forebrain NPCs

We first examined the expression of Yap1 in the early (E11 and E12) telencephalic and diencephalic walls. Observation of sections immunostained with anti-Yap1 antibody at low magnification revealed intense Yap1 immunoreactivity in the NE and VZ of both regions, which were also positive for Pax6 (Fig. 1a, b). In the neuronal zone forming outside the VZ, Yap1 immunoreactivity was only faintly detected. In individual E11, E14, and E16 telencephalic cells, single-cell transcriptome analysis, which can discriminate among cells of distinct differentiation status [43, 44], revealed prominent *Yap1* expression in undifferentiated NPCs but not in neurons (Fig. 1i), confirming our immunohistochemical finding. At a higher magnification (Fig. 1c–h), Yap1 immunoreactivity was most clearly observed in the cytoplasm of the pseudostratified somata of NPCs, as well as in NPCs’ radial processes extending basally (towards the pial surface). But, it was also detected in the nucleus (Fig. 1d, d′, e), similar to a pattern previously observed in the neural tube at E8.5 and E10.5 [26]. This nuclear Yap1 localization, a sign of Yap1’s contribution to proliferation [25–27], is consistent with previously reported Yap1’s roles in NPCs [24, 28–32]. Nuclear Yap1 immunorectivity was not homogeneous in the NE/VZ (i.e. some nuclei were more strongly stained than others, Fig. 1d′), although the reason for this remains unknown.

### Yap1 or Tead Enhances Stem-Like Behaviors of NPCs in Both the Dorsal Telencephalon and the Dorsal Diencephalon

To increase Yap1 expression in the dorsal diencephalic or telencephalic walls, we used the Yap1-S112A mutant, previously shown in NIH3T3 cells to induce proliferation, resulting in denser confluence than that achieved by Yap1 without mutations [26, 33]. We electroporated E10 mice with a GFP-tagged Yap1-S112A or a control GFP vector. After 1 day (at E11), we compared anti-Yap1 immunoreactivity between un-electroporated, control GFP–electroporated, and Yap1-S112A–electroporated cells. Yap1 immunoreactivity was more intense in the Yap1-S112A–electroporated NPCs (both cytoplasmic and nuclear) than in un-electroporated or control GFP–electroporated NPCs in both diencephalon (Fig. 2b) and telencephalon (data not shown), confirming that our IUE successfully increased the expression of Yap1 in a region-specific manner.

We next sought to evaluate the stem-like (i.e. self-renewing) status of the Yap1-S112A–electroporated NPCs. Consistent with previous studies revealing the formation of a hypertrophic VZ in the Yap-overexpressed telencephalon [30, 31], our comparison of the proportion of the total GFP^+^ cells that were also positive for Pax6, a transcription factor expressed in stem-like NPCs [8, 43, 44] to control the balance between their proliferative and undifferentiated (VZ-remaining) status and differentiation [45, 46], between control and Yap1-S112A IUE telencephalons (analyzed at E13) revealed that the descendant cells of the Yap1-S112A–electroporated cells were more frequently Pax6^+^ (61%) than those of control GFP–electroporated cells (48%) (p = 0.0012) (Fig. 2a, c), suggesting that Yap1-S112A–electroporated NPCs engaged in self-renewal (i.e., they divided to give rise to stem-like daughter cells) to a greater extent than the controls. We also electroporated E10 telencephalons with activator-modified Tead2 (Tead2-VP16), previously shown to mediate Yap-dependent enhancement of proliferation in both NIH3T3 cells [26] and NPCs [24, 32]. The frequency of Pax6 expression in GFP^+^ cells (%Pax6^+^/GFP^+^) in Tead2-VP16 IUE was greater (67%) than in control GFP IUE (48%) (p = 0.0006) (Fig. 2c), as also observed in the Yap1-S112A IUE experiments.

Also in the diencephalon, %Pax6^+^/GFP^+^ increased following introduction of Yap1-S112A (78% compared to 55% in control, p = 0.0022) or Tead2-VP16 (82%, p = 0.0043) at E13. The increase in %Pax6^+^/GFP^+^ from the control level was comparable to that observed in the telencephalon (+27% by Yap1-S112A and +40% by Tead2-VP16 in the telencephalon; +42% by Yap1-S112A and +49% by Tead2-VP16 in the diencephalon). Because the stem-like characteristics of NPCs have not been as carefully analyzed as those of cells in the telencephalon, we performed a BrdU-based pulse-chase experiment [3, 12, 14] starting at E11. At E12 (i.e., 24 h after administration of a BrdU pulse at E11 and 48 h after IUE at E10) (Fig. 2a, d), the frequency of expression of p27 (Cdkn1b) (a cell-cycle inhibitor, expressed in cells that have exited the cell cycle [43]) among all GFP^+^BrdU^+^ cells was significantly lower in Yap1-S112A IUE (5%) (p = 0.0022) and Tead-VP16 IUE (0%) (p = 0.0043) than in control IUE (20%) (Fig. 2d). Furthermore, %Pax6^+^/GFP^+^BrdU^+^ was significantly higher in Tead-VP16 IUE (72%) (p = 0.0002) than in control IUE (58%). Yap1-S112A IUE also exhibited a significantly higher %Pax6^+^/GFP^+^BrdU^+^ (74%) (p = 0.0047) (Fig. 2d). These results indicate that both Yap1-S112A and Tead2-VP16 electroporated at E10 subsequently induced the diencephalic NPCs to choose a division mode biased towards proliferation rather than differentiation (i.e., to remain as stem-like cells). Taken together, these results suggest that excessive expression of Yap1-S112A or Tead2-VP16 in the early telencephalon and diencephalon enhances in stem-like characteristics of NPCs, thereby inducing cells to stay in the VZ.

### NPC Heterotopias Form More Frequently in the Dorsal Diencephalon than in the Dorsal Telencephalon

We next asked whether telencephalic or diencephalic VZs exhibiting an elevated tendency toward self-renewal might form NPC heterotopias, as previously observed in the neural tube [24]. By E13, the Yap/Tead-overexpressing dorsal diencephalic walls frequently formed massive NPC heterotopias (Fig. 3) [extra-VZ NPCs were present in 61% in Yap1-S112A IUE (p = 0.0022) and 63% in Tead2-VP16 IUE (p = 0.0043), versus 13% in control IUE]. Each heterotopia, 150–200 µm away from the apical/ventricular surface and within the TUJ1^+^ neuronal territory, consisted of many GFP^+^TUJ1^−^ somata (each containing the Pax6^+^ nucleus) (10–50 per 16-µm-thick section), clustered to become ~50 µm in aggregate diameter. Up to 63% of GFP^+^Pax6^+^ cell nuclei were outside the VZ, and formation of heterotopia was very frequent (i.e., in most Yap1-S112A or Tead2-VP16 IUE cases). GFP immunostaining suggested that most (if not all) of these heterotopically aggregated NPCs had lost connections to the apical surface. In the control IUE, by contrast, GFP^+^ cells that were judged to have lost apical attachment were TUJ1^+^ (Pax6-negative) neurons.

Occasionally in Yap/Tead-overexpressing embryos, Pax6^+^ but GFP-negative nuclei/somata were observed in or near the heterotopic NPC aggregates (~6% of all heterotopic Pax6^+^ cells). Although some GFP-negative Pax6^+^ cells were observed in the control IUE cases, they were distributed within 120–130 µm from the apical surface and close to the basal border of the VZ (Fig. 3c). GFP-negative Pax6^+^ cells in the Yap/Tead-overexpressing cases were observed in more basal portions (150–200 µm from the apical surface), near the GFP^+^Pax6^+^ cells massively occupying such far-basal regions. Therefore, it is possible that not only GFP^+^ electroporated cells or their descendant cells, but also a minor fraction of the surrounding non-electroporated (GFP^−^) VZ cells, were detached from the apical surface, presumably due to mechanical effects near the apical surface, such as overcrowding [11, 14, 47].

**Fig. 5:**
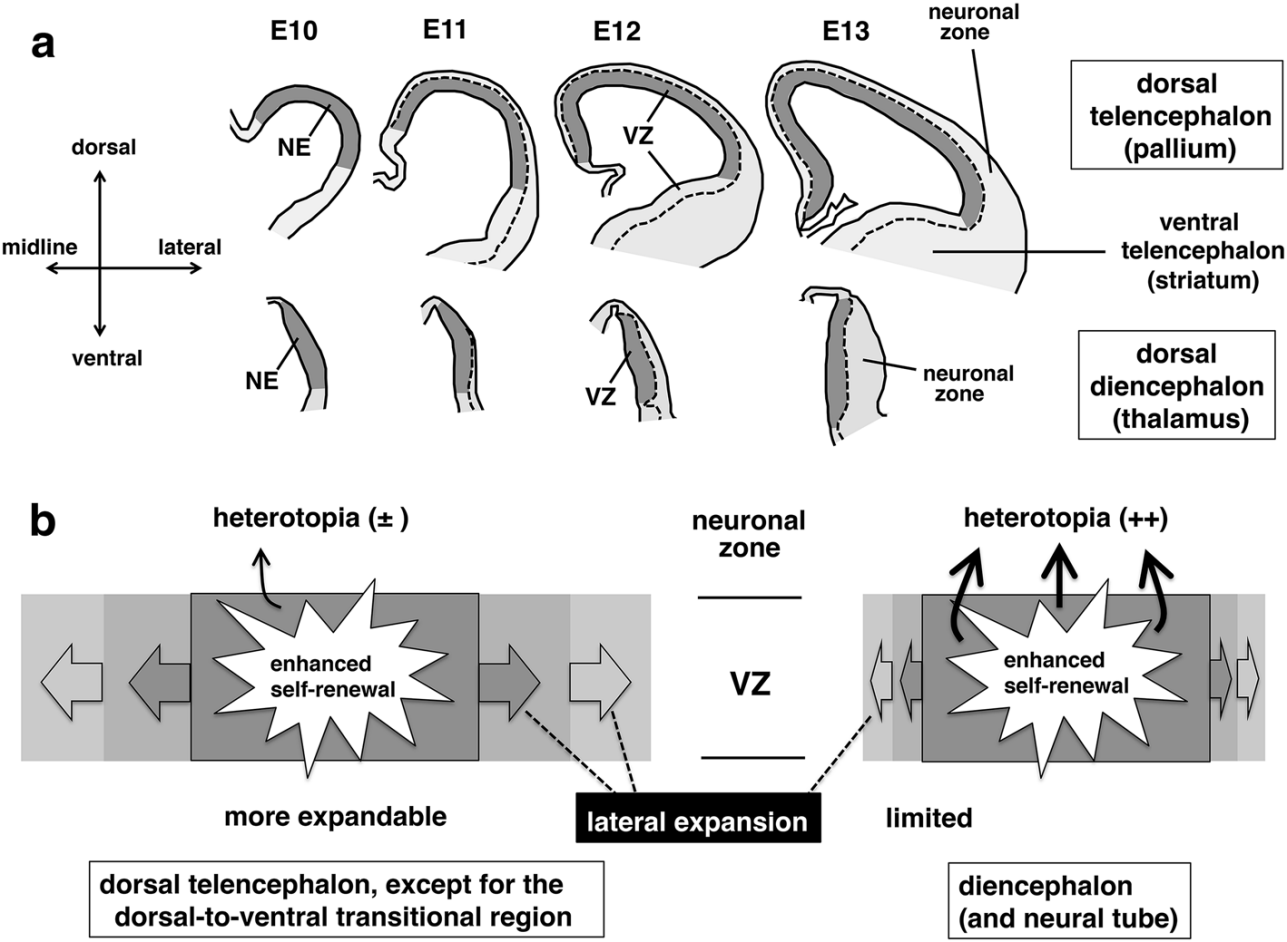
Model of differential heterotopia formation by Yap1/Tead-overexpressing hyper-self-renewing dorsal diencephalic and telencephalic NPCs, possibly depending on tissue-level permissiveness for lateral expansion of the NE/VZ. A trace of the growing telencephalic and diencephalic walls (**a**), reproduced based on Altman and Bayer [40], shows that by E13, the telencephalic NE/VZ laterally expands such that it becomes three times broader than at E10, whereas the diencephalic NE/VZ cannot expand so extensively along the dorso-ventral axis. Such differential tangential expandability could explain the differential heterotopia formation by NPCs (**b**)

In dorsal telencephalon subjected to overexpression of Yap1-S112A or Tead-VP16, NPC heterotopias were observed much less frequently [extra-VZ NPCs were observed in 6% of Yap1-S112A IUE (p = 0.73) and 8% of Tead2-VP16 IUE (p = 0.27), versus 5% of control IUE] (Fig. 4a–c), and clear aggregates, which were frequently observed in diencephalic Yap1-S112A or Tead2-VP16 IUE cases (>30 µm in diameter), were not observed. However, we observed relatively large (~50 µm) NPC heterotopias only when the most ventrolateral part of the dorsal telencephalic VZ was targeted by IUE (Fig. 4d). This specific region may be under a physical constraint due to the neighboring (but geometrically distinct) VZ of the ventral telencephalon (future striatum) (schematically illustrated in Fig. 5a). Although the apical surface of the dorsal telencephalon is concave, that of the ventral telencephalon is convex, making the dorsal-to-ventral transitional region of the VZ bend abruptly in a hairpin loop. In our previous experiments in which Wnt3a was overexpressed in the dorsal telencephalic VZ, this most ventrolateral part was most extensively thickened along the apicobasal axis, and formed frequent large NPC heterotopias, in contrast to other dorsal telencephalic VZ regions that seemed to have freely expanded tangentially (along the dorso-ventral axis) without apicobasal thickening [14]. Physiological non-surface mitoses in the dorsal telencephalon are observed earliest, and most frequently, in this ventrolateral end region [48].

Although the precise mechanism underlying the differential heterotopia formations between the diencephalic and telencephalic NPCs under enhanced self-renewal due to artificial activation of Yap1/Tead remains unknown, our results suggest that NPC heterotopias may occur as a reflection of region-specific three-dimensional conditions. As schematically (but semi-quantitatively) traced in Fig. 5a, the telencephalic and diencephalic VZs differ in regard to their lateral expandability during early embryonic days. The lateral extension of the dorsal diencephalon is much smaller than that of the dorsal telencephalon, which forms a large vesicle/hemisphere [40, 49]. Such differential tangential expandability of VZ (i.e., tissue-level permissiveness for lateral expansion of the NE/VZ: telencephalon > diencephalon) could explain the differential NPC heterotopia formation (telencephalon < diencephalon) that we observed in our Yap1/Tead experiments.

In line with this model, we have observed that E11 or E12 dorsal diencephalic walls to which Yap1/Tead had been introduced at E10 did not form heterotopias when sliced and cultured in collagen gel (Saito et al., unpublished observation). Although collagen gel gently holds embedded slices to assist our time-lapse observations, it allows slices to grow almost freely, without physically restricting slices’ expansion and thickening. VZ in slice cultured brain walls may be less laterally compressed than in vivo (i.e. permissiveness to VZ’s lateral expansion is greater in slice culture than in vivo), thereby failing to reproduce the in vivo (heterotopia-forming) behaviors of the Yap1/Tead-introduced dorsal diencephalic VZ cells.

Excessive compression at/near the apical surface is involved in epithelial cells’ detachment from the apical surface [11, 14, 47]. Limited lateral expandability of the VZ (Fig. 5b), which is more likely to arise in the diencephalon and the neural tube than in the telencephalon, may thus act as an inducer for NPCs to leave the apical surface. These results provide a basis for future studies aimed at revealing how Yap1/Tead might act in NPCs to sense and/or respond to such hypothetical physical/mechanical limitations on the lateral expansion of VZ, and thereby contribute to heterotopia formation.

## Abbreviations

BrdU: Bromodeoxyuridine
CNS: Central nervous system
E: Embryonic day
IKNM: Interkinetic nuclear migration
IUE: In utero electroporation
NE: Neuroepithelium
NPC: Neural progenitor cell
Tead: TEA domain transcription factor
VZ: Ventricular zone
Yap1: Yes-associated protein 1
